# Primary diffuse large B-cell lymphoma of the breast: A retrospective study of outcomes and insulin resistance

**DOI:** 10.15537/smj.2023.44.1.20220677

**Published:** 2023-01

**Authors:** Weiling Zhou, Guodong Yu, Lihong Liu, Qian Gao, Lei Feng, Yuan Wang

**Affiliations:** *From the Department of Endocrine and Metabolic Diseases (Zhou, Gao, Feng, Wang), from the Department of Hematology (Liu), The Fourth Hospital of Hebei Medical University, Shijiazhuang, and from the Department of Hepatobiliary Surgery (Yu), Affiliated Hospital of Hebei University, Baoding, China.*

**Keywords:** breast, insulin resistance, diffuse large B-cell lymphoma, therapy, prognosis

## Abstract

**Objectives:**

To investigate the clinicopathological features, insulin resistance (IR) status, and the outcomes of populations with diffuse large B-cell lymphoma (DLBCL) of the breast.

**Methods:**

This study was carried out at Department of Haematology, the Fourth Hospital of Hebei Medical University, Shijiazhuang, China, that included 32 patients treated form January 2009 to June 2020. The primary endpoints of the study were their survival time.

**Results:**

There were 32 patients in the study. A total of 18 (56.2%) patients had IR. In terms of treatment, 31.2% were treated with surgery, most (93.8%) received chemotherapy, and 25% received radiotherapy and intrathecal therapy. Univariate analysis indicated the patients with stages III-IV, B symptoms, tumour recurrence, PAX5 positivity, and c-MYC positivity showed a shorter survival time (*p*<0.05). The overall survival and progression-free survival (PFS) rates in IR group were shorter than those without IR, but there was no statistical difference (*p*>0.05). Multivariate analysis indicated that tumour recurrence shortened the 5-year PFS of the patients (*p*=0.037).

**Conclusion:**

Primary DLBCL of the breast was very rare; more than half of the cases had IR, but IR did not affect their survival.

**D**iffuse large B-cell lymphoma (DLBCL) is the malignancy of the blood system, accounting for approximately 24-30% of Non-Hodgkin’s lymphoma (NHL).^1^ Primary breast lymphoma is extremely rare, accounting for approximately 0.5% malignancies of the breast and 1% of all NHL patients.^2^ Insulin resistance (IR) meas the inefficiency of insulin in promoting glucose ingestion and utilization. Hyperinsulinemia occurs when the body secretes excess insulin in compensation, which often leads to diabetes and metabolic syndrome. Insulin resistance was related with a higher incidence of breast cancer and post-breast cancer, all-cause mortality, which may be attributed to the direct cancer promoting effect of insulin on tumor cells.^3,4^ In our hospital, we found that more than 50% of the patients with DLBCL of the breast had IR before therapy; when we reviewed the patients treated over the past 11 years, which was surprising. Whether IR contributes to the prevalence of DLBCL of the breast or influences its prognosis is still unknown. Since primary DLBCL of the breast is very rare and there is little published research. We retrospectively analyzed the detailed clinicopathological features and prognosis of 32 cases with DLBCL of the breast.

## Methods

We included 32 patients diagnosed with primary DLBCL of the breast at the Fourth Hospital of Hebei Medical University, Shijiazhuang, China, from January 2009 to June 2020. The exclusion criterion was from other site metastasized to the breast. Following the principles of the Helsinki Declaration and the Medical Ethics Committee of the hospital, therefore, this study was approved.

The patients’ date were gathered from inpatient medical records, outpatient visits, and telephone follow-up until July 2020. The data included age, gender, tumour size, primary site, pathological diagnosis, international prognostic index (IPI), stage and B symptoms, fasting plasma glucose (FPG) values, immunohistochemistry, comorbidities, family history of cancer, progression-free survival (PFS), and overall survival (OS).

Specimen of blood were obtained before therapy when the patients diagnosed with DLBCL of the breast. After an overnight fast, 5 mL venous blood samples from all 32 subjects were collected in tubes containing ethylenediaminetetraacetic acid (EDTA) anticoagulation, centrifuged to separate the plasma, and then stored at -20°C until used in assays. The concentrations of serum glucose were measured by glucose oxidase method (Makerbio, China). Insulin levels were measure by electrochemiluminescence method (Roche Diagnostics GmbH, Germany). Homeostasis model assessment (HOMA) value of >2.7 defined as IR, calculated by the formula (FPG [mmol/l]×fasting insulin [μU/ml])/22.5.^5^

Data on the treatment regimens of all the patients were collected, including surgery, chemotherapy drugs and number of cycles, radiotherapy, stem cell transplantation, and intrathecal therapy. The definitions and consensus response evaluation criteria of lymphoma from the International Working Group (RECIL 2017) were used.^6^

Overall survival defined from the date of diagnosed as DLBCL to death from any reason or loss to follow-up. Progression-free survival defined from the date of diagnosed as DLBCL to relapse, disease progression or last follow-up.

### Statistical analysis

Data were statistically analysed by using the Statistical Package for the Social Sciences, version 22.0 (IBM Corp., Armonk, NY, USA). Continuous variables were shown as medians and ranges, and frequencies and percentages were shown for the categorical variables. Multivariate analysis used Cox proportional risk model. A *p*-value of <0.05 was considered as statistical differences.

## Results

The study contained 32 people with DLBCL of the breast, and their mean age was 59.13±13.27 years (range: 27-84). Of these patients, 1 (3.1%) was male and 31 (96.9%) were female. All patients presented with solitary masses, occurring in 16 (50%) cases within the right breast and in 2 (6.2%) cases bilaterally. The average tumour size was 4.15 cm (range: 1.0-10.18), 13 (40.6%) patients had tumour (≥5 cm). A total of 20 (62.5%) patients developed metastases, including 16 lymph node metastases. A total of 25 (78.1%) patients had comorbidities (diabetes, hypertension, hepatitis, cerebrovascular disease, coronary heart disease, and uterine fibroids), 4 patients had family genetic history of cancer, while one patient had family genetic history of the breast cancer. A total of 11 (34.4%) patients experienced a recurrence of primary DLBCL of the breast during the study period. There were 18 (56.2%) patients with IR (Table 1).

**Table 1:** - Clinical characteristics and univariate analysis of 32 patients with diffuse large B-cell lymphoma of the breast.

Variables	All (n=32)	5 years progression-free survival		5 years overall survival	
		Precentages	*P*-values	Precentages	*P*-values
***Age at diagnosis***	59.13±13.27				
≥60 years	18 (56.3)	81%	0.629	81%	0.738
<60 years	14 (43.7)	55%		63%	
***Gender***					
Male	1 (3.1)	--	--	--	--
Female	31 (96.9)				
***Primary site***					
Left	14 (43.8)	69%	0.171	68%	0.129
Right	16 (50.0)	80%		82%	
Bilateral	2 (6.2)	50%		50%	
***Tumor size***	4.15±2.64				
≥5 cm	13 (40.6)	74%	0.129	72%	0.172
<5 cm	19 (59.4)	66%		67%	
***Ann-Arbor stage***					
III-IV	13 (40.6)	61%	0.047*	61%	0.031*
I-II	19 (59.4)	75%		71%	
***IPI score***					
3-5 score	26 (81.2)	67%	0.445	67%	0.360
0-2 score	6 (18.8)	71%		72%	
***B symptoms***					
Yes	7 (21.9)	52%	0.084	52%	0.026*
No	25 (78.1)	78%		79%	
***Pathological diagnosis***					
GCB	7 (21.9)	100%	0.164	100%	0.110
Non-GCB	25 (78.1)	64%		60%	
***Metastasis***					
Yes	20 (62.5)	64%	0.235	62%	0.204
No	12 (37.5)	88%		89%	
***Recurrence***					
Yes	11 (34.4)	30%	<0.001*	40%	0.001*
No	21 (65.6)	100%		100%	
***Outcome***					
Alive	26 (81.3)	--	--	--	--
Die	6 (18.7)				
***Comorbidities***					
Yes	25 (78.1)	69%	0.468	69%	0.457
No	7 (21.9)	73%		73%	
Insulin (uU/ml)	10.54±6.10	--	--	--	--
FPG (mmol/L)	5.93±2.50	--	--	--	--
***HOMA-IR***	2.45±0.74				
>2.7	18 (56.2)	63%	0.864	66%	0.885
≤2.7	14 (43.8)	78%		79%	

Values are presented as numbers and prescentages (%).

* *P*-value of <0.05 was significant. IPI: international prognostic index, GCB: germinal center B-cell, FPG: fasting plasma glucose, HOMA-IR: homeostasis model assessment of insulin resistance

The follow-up period ended in July 2020. Among the 32 patients, 26 (81.3%) were alive, and 6 (18.7%) had died. The average follow-up time was 39.66±35.43 months (range: 10-144). The PFS and OS in 3 years were 82%, 44% and in 5 years were 83% and 49%. Univariate analysis indicated the 5-year survival time of populations with stages III-IV, B symptoms, and tumour recurrence during the study period was shorter (*p*<0.05). Cox multivariate analysis indicated PFS of patients with tumour recurrence was shorter (*p*=0.037; Table 2).

**Table 2:** - Multivariate analysis of prognostic factors for **progression-free survival** and **overall survival** in 32 patients with diffuse large B-cell lymphoma of the breast

Factors	Progression-free survival			Overall survival		
	HR	95% CI	*P*-values	HR	95%CI	*P*-values
Ann Arbor stage (III-IV, I-II)	2.18	0.20-23.33	0.519	3.75	0.39-36.15	0.25
B symptoms (Yes, No)	3.34	0.55-20.46	0.19	3.60	0.54-24.08	0.19
Recurrence (Yes, No)	10.99	1.16-104.37	0.037*	7.59	0.87-66.13	0.066

* P-value of <0.05 was significant. HR: hazard ratio, CI: confidence interval

**Figure 1:**
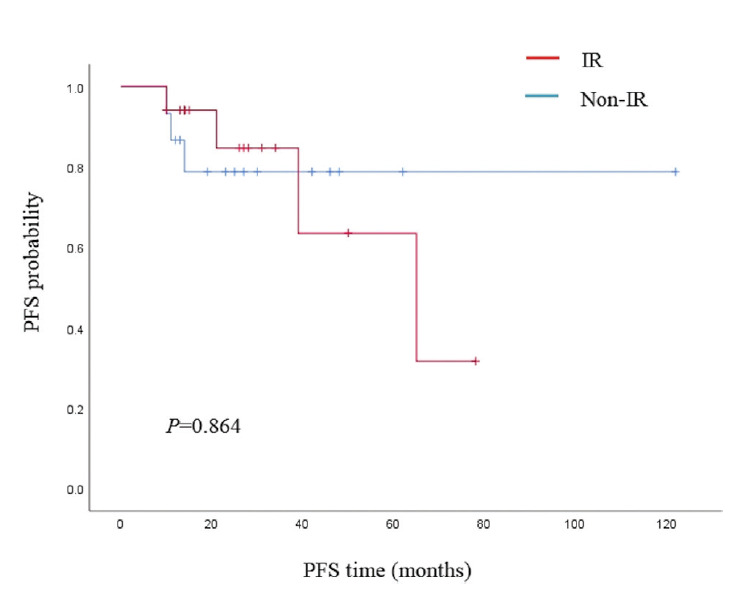
- Progression-free survival of the insulin resistance group and non-insulin resistance group. PFS: progression-free survival, IR: insulin resistance, non-IR: non-insulin resistance

**Figure 2:**
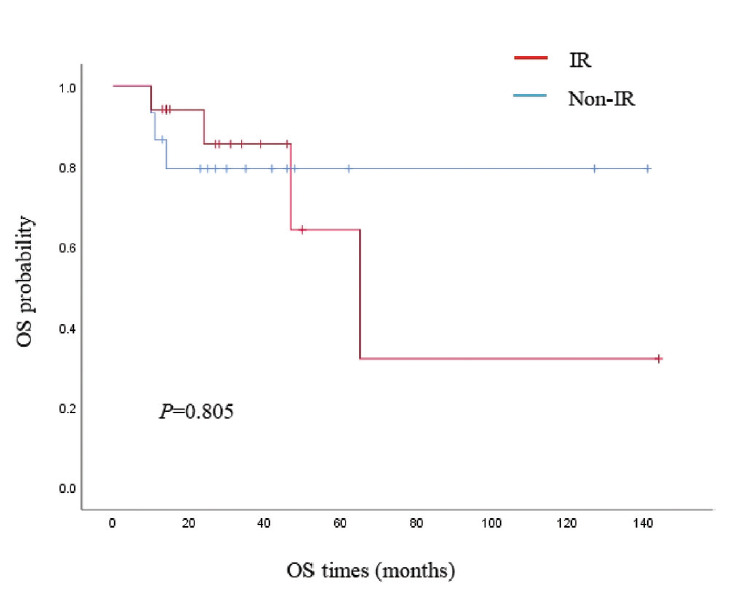
- Overall survival of the insulin resistance group and non-insulin resistance group. OS: overall survival, IR: insulin resistance, non-IR: non-insulin resistance

The patients with IR had a poorer prognosis than those without IR, but there was no statistical difference (*p*>0.05; Figures 1&2).

In all patients, the pathological diagnosis of primary DLBCL of the breast was confirmed by immunohistochemistry. The Ki-67 immunostaining proliferation index was ≥90% in 9 people, Bcl-2 positivity was found in 24 patients, Bcl-6 positivity was found in 21 patients, C-MYC positivity was found in 25 patients, and CD79α positivity was found in 7 patients. A total of 10 patients expressed PAX5, 5 patients expressed CD5, and 29 patients expressed MUM1. Univariate analysis showed that the C-MYC-positive and Pax5-positive patients had poor 5-year OS and PFS (*p*<0.05; Table 3).

**Table 3:** - Immunohistochemistry in 32 patients with primary diffuse large B-cell lymphoma of the breast.

Immunohistochemistry	All	5 years progression-free survival		5 years overall survival	
		Precentages	*P*-values	Precentages	*P*-values
***Ki-67***					
≥90%	8 (25.0)	85%	0.714	86%	0.670
<90%	24 (75.0)	69%		68%	
***BCL-2***					
Positive	24 (75.0)	74%	0.455	72%	0.629
Negative	8 (25.0)	73%		75%	
***BCL-6***					
Positive	21 (65.6)	89%	0.072	90%	0.107
Negative	11 (34.4)	42%		50%	
***C-MYC***					
Positive	25 (78.1)	18%	0.001*	29%	0.004*
Negative	7 (21.9)	48%		48%	
***CD79***					
Positive	7 (21.9)	69%	0.438	71%	0.583
Negative	25 (78.1)	73%		70%	
***PAX-5***					
Positive	10 (31.2)	26%	0.001*	18%	0.001*
Negative	22 (68.8)	94%		95%	
***MUM1***					
Positive	29 (90.6)	73%	0.617	73%	0.547
Negative	3 (9.4)	67%		67%	
***CD5***					
Positive	5 (15.6)	100%	0.279	100%	0.228
Negative	27 (84.4)	68%		66%	

Values are presented as numbers and prescentages (%).

* Significant (*p*<0.05).

Among the 32 patients, 10 (31.2%) underwent mastectomy, and 30 (93.8%) underwent chemotherapy, of whom 28 (87.5%) underwent ≥4 chemotherapy cycles, 8 (25%) underwent radiotherapy, 8 (25%) received intrathecal therapy, and 2 (6.2%) received stem cell transplantation. After treatment, 21 (65.6%) achieved complete remission, 3 (9.4%) achieved partial remission, and 8 (25%) achieved patients progressive disease. Univariate analysis indicated the 5-year OS and PFS rates were higher in the CR population (*p*<0.05; Table 4).

**Table 4:** - The treatment of patients with primary diffuse large B-cell lymphoma of the breast (N=32).

Treatments	All	5 years progression-free survival		5 years overall survival	
		Precentages	*P*-values	Precentages	*P*-values
***Mastectomy***					
No	22 (68.8)	59%	0.917	60%	0.984
Yes	10 (31.2)	77%		79%	
***Chemotherapy***					
Yes	30 (93.8)	--	--	--	--
No	2 (6.2)				
***Cycles of chemotherapy***					
≥4cycles	28 (87.5)	67%	0.303	68%	0.301
<4cycles	4 (12.5)	100%		100%	
***Radiotherapy***					
Yes	8 (25.0)	69%	0.321	73%	0.593
No	24 (75.0)	75%		72%	
***Intrathecal therapy***					
Yes	8 (25.0)	85%	0.699	86%	0.703
No	24 (75.0)	69%		70%	
***Transplantation***					
Yes	2 (6.2)	--	--	--	--
No	30 (93.8)				
***Responses***					
CR	21 (65.6)	95%	0.007*	95%	0.015*
PR	3 (9.4)	33%		33%	
SD	0 (0.0)	--		--	
PD	8 (25.0)	32%		54%	

Values are presented as numbers and prescentages (%).

* Significant (*p*<0.05). CR: complete remission, PR: partial remission, SD: stable disease, PD: patients progressive disease

## Discussion

This 11-year retrospective study included 32 people with rare DLBCL of the breast. Their pathological characteristics, treatment, and outcomes were analyzed in detail. This study found that patients with DLBCL of the breast often had IR. Average age of the cases was 59.13 years, while there was only one male among the 32 patients in this study, that was similar to Genco et al’s study.^7^ Our results indicated that the 5-year PFS in patients with DLBCL of the breast was 44% and the OS was 49%. This study also showed that 5 year survival time was not related to age, tumour site, tumour size, IPI score, B symptoms, pathological origin, metastasis, comorbidities, or IR. However, other studies have shown that peoples with breast DLBCL with a tumour size more than 5 cm had a poor prognosis.^8^ The differences between outcomes of the study and other studies perhaps were related to the study patients size.

This study also analyzed in details the relationship between the immunohistochemistry and the patients’ 5-year survival rates. The results showed that PAX5 positivity and C-MYC positivity were associated with poor prognoses. The PAX5 gene, known as B cell-specific activator protein, plays a significant role in the proliferation of B-cells, isotype switching, immunoglobulin gene transcription, and cell differentiation. In lymphomas, the gene of the C-MYC abnormal express were almost limited to B-cell lymphomas, including rearrangements and amplifications.^9^ Translocations of BCL2, C-MYC, and BCL3 or BCL6 genes were crux characteristics of double/triple hit lymphoma (DHL/THL).^10^ Double/triple hit lymphoma of the patients showed short survival time with present treatment.^11^ Due to the long time span of this study, fluorescence in situ hybridization (FISH) or chromosome examinations of specimen were not carried out, so DHL/THL lymphoma models were not included.

Because primary DLBCL of the breast is extremely rare, many patients were misdiagnosed with breast cancer and underwent surgical treatment. In this study, 10 (31.2%) underwent mastectomy, which did not significantly improve their survival. Some else therapy of the patients included chemotherapy, radiotherapy, intrathecal therapy, and stem cell transplantation. Although these treatments improved survival time to some extent, the results were not statistically significant, similar to the findings of Luo et al^12^ and Lamy et al.^13^ Therefore, in future clinical work, surgical treatment should not be carried out blindly for breast masses. Preoperative pathological diagnosis should be made clear, and specific treatment plan should be determined according to the pathological results.

Interestingly, when we collated the relevant cases, we found that patients with DLBCL of the breast often had IR, which was not seen in previous reports. In this study, 18 (56.2%) developed IR, and 9 of these had diabetes. Some studies had shown that IR was a risk factor of the breast cancer, and it may be considered as a factor for these populations.^14,15^ Insulin resistance was a factor mediating the association between race and poor breast cancer prognosis in a multicentre, cross-sectional study in the United States.^16^ Raised Notch signalling in mice contributes to white adipose tissue blocked from expanding, which may leads to IR.^17^ Chronic over-activation of Notch signalling impairs insulin sensitivity, leading to IR.^18^ This study found that the IR group had a higher body mass index, but there was no statistical difference compared with the non-IR group, it may have something to do with the small sample size. The specific mechanism that led to the higher rate of IR in the patients in this study was unclear, and it may be related to obesity and diabetes. The survival time of the populations with IR was poor than those without IR, but there was no statistical difference. As these results probably were related to the little sample capacity, further studies with larger samples size are demanded to verify this conclusion.

### Study limitations

It was a retrospective study rather than a prospective study and it was a single-centre study and the number of cases was relatively small.

In conclusion, this study described pathological characteristics, treatment, and prognosis of the populations with DLBCL of the breast in details and we propose that those patients often had IR, but IR did not affect their survival. A large data volume, multi-center study is needed to verify this results in future studies.
